# FOXM1 is a downstream target of LPA and YAP oncogenic signaling pathways in high grade serous ovarian cancer

**DOI:** 10.18632/oncotarget.4280

**Published:** 2015-06-13

**Authors:** Qipeng Fan, Qingchun Cai, Yan Xu

**Affiliations:** ^1^ Department of Obstetrics and Gynecology, Indiana University School of Medicine, Indianapolis, IN 46202, USA

**Keywords:** EOC (epithelial ovarian cancer), FOXM1, HGSC (high grade serous ovarian cancer), LPA (lysophosphatidic acid), YAP (yes-associated protein)

## Abstract

Lysophosphatidic acid (LPA), a prototypical ligand for G protein coupled receptors, and Forkhead box protein M1 (FOXM1), a transcription factor that regulates expression of a wide array of genes involved in cancer initiation and progression, are two important oncogenic signaling molecules in human epithelial ovarian cancers (EOC). We conducted *in vitro* mechanistic studies using pharmacological inhibitors, genetic forms of the signaling molecules, and RNAi-mediated gene knock-down to uncover the molecular mechanisms of how these two molecules interact in EOC cells. Additionally, *in vivo* mouse studies were performed to confirm the functional involvement of FOXM1 in EOC tumor formation and progression. We show for the first time that LPA up-regulates expression of active FOXM1 splice variants in a time- and dose-dependent manner in the human EOC cell lines OVCA433, CAOV3, and OVCAR5. G_i_-PI3K-AKT and G_12/13_-Rho-YAP signaling pathways were both involved in the LPA receptor (LPA_1–3_) mediated up-regulation of FOXM1 at the transcriptional level. In addition, down-regulation of FOXM1 in CAOV3 xenografts significantly reduced tumor and ascites formation, metastasis, and expression of FOXM1 target genes involved in cell proliferation, migration, or invasion. Collectively, our data link the oncolipid LPA, the oncogene YAP, and the central regulator of cell proliferation/mutagenesis FOXM1 in EOC cells. Moreover, these results provide further support for the importance of these pathways as potential therapeutic targets in EOC.

## INTRODUCTION

Despite the pathological and genetic heterogeneity, high-grade serous ovarian carcinomas (HGSC) share common molecular alterations. In addition to TP53, integrated HGSC studies identified that forkhead box M1 (FOXM1) transcription factor network is significantly altered in 84% of HGSC cases [1, 2].

The FOXM1 protein is a proliferation-specific transcription factor that plays a key role in controlling expression of cell cycle genes essential for tumorigenesis [3]. Overexpression of FOXM1 and its potential correlations to worse prognosis and/or drug-resistance in epithelial ovarian cancer (EOC) have been reported by several labs [1, 2, 4–6]. Lok *et al* have shown that the overexpressed phospho-ERK and FOXM1 are correlated well and they are significantly correlated to HGSC with aggressive behavior [7]. In addition, inhibition of FOXM1 expression by either thiostrepton, a selective FOXM1 inhibitor, which may inhibit FOXM1 at the transcriptional, post-transcriptional, as well as its promoter binding levels [8–11], or U0126 could significantly impair FOXM1-mediated oncogenic capacities [12]. Moreover, overexpression of FOXM1 predicts poor prognosis and FOXM1 promotes proliferation, migration and invasion in the EOC cell line HO-8910 *in vitro* [5, 13]. However, the concept of targeting FOXM1 in EOC needs to be tested further since the mouse model and reagents used may improperly address the targeting issues related to FOXM1, due to the potential off-target effects induced by the inhibitor, the non-HGSC nature of the cell line used (A2780 or SKOV3), or the route of injection (s.c.) [5, 14]. In particular, FOXM1’s role in endothelial cells and other host cells as reported [15] may complicate the anticancer strategies targeting FOXM1 and suggests that specific tumor cells targeting is necessary.

Since FOXM1 displays a strictly proliferation-specific expression pattern, its expression is up-regulated by proliferation signals, but down-regulated by anti-proliferation signals, in particular, proto-oncoproteins (such as AKT and Ras) and growth factors and/or their receptors [such as epidermal growth factor receptors (EGFRs) and hepatocyte growth factor receptor (HGFR)] [3]. Interestingly, YAP (yes-associated protein) has been shown to be able to directly induce the transcription of CCND1 and FOXM1 via the YAP-TEAD binding site in the FOXM1 promoter in malignant mesothelioma (MM) cells [16]. Surprisingly, there is no follow-up publication in YAP-regulated FOXM1 in any other cell types, in spite of the clear emerging attention to the roles of the Hippo-YAP pathway in various cancers [17–19]. In addition, the potential regulation of FOXM1 by G protein coupled receptors (GPCRs) and their ligands have not been demonstrated in any cell type.

We and others show that lysophosphatidic acid (LPA), a small bioactive phospholipid, is elevated in the blood of EOC patients [20] and it stimulates cell proliferation, migration, invasion, angiogenesis, and tumor metastasis of EOC [21–23]. LPA has been considered as an oncolipid and an important target for EOC treatment [23–25]. LPA mediated its functions via its G protein coupled receptor (GPCR) LPA_1–6_ [26]. LPA_1–3_ receptors are functionally involved in EOC cells [18, 27–30]. We have recently shown that LPA dose- and time-dependently induced YAP dephosphorylation (dpYAP) in human EOC cell lines OVCA433, OVCAR5, CAOV3, and Monty-1, accompanied by increased YAP nuclear translocation and activation [18].

To determine whether LPA itself, which is a prototype for GPCR signaling, is able to regulate FOXM1 in cells, we conducted both *in vitro* and *in vivo* mechanistic and functional studies in several EOC cell lines. In addition, we tested the functional involvement of FOXM1 *in vivo* using a xenograft mouse model. Our work has functionally linked the oncolipid LPA, the oncogene YAP, and the central regulator of cell proliferation/mutagenesis FOXM1 in EOC cells.

## RESULTS

### LPA induced FOXM1 up-regulation in EOC cells

**Figure 1:**
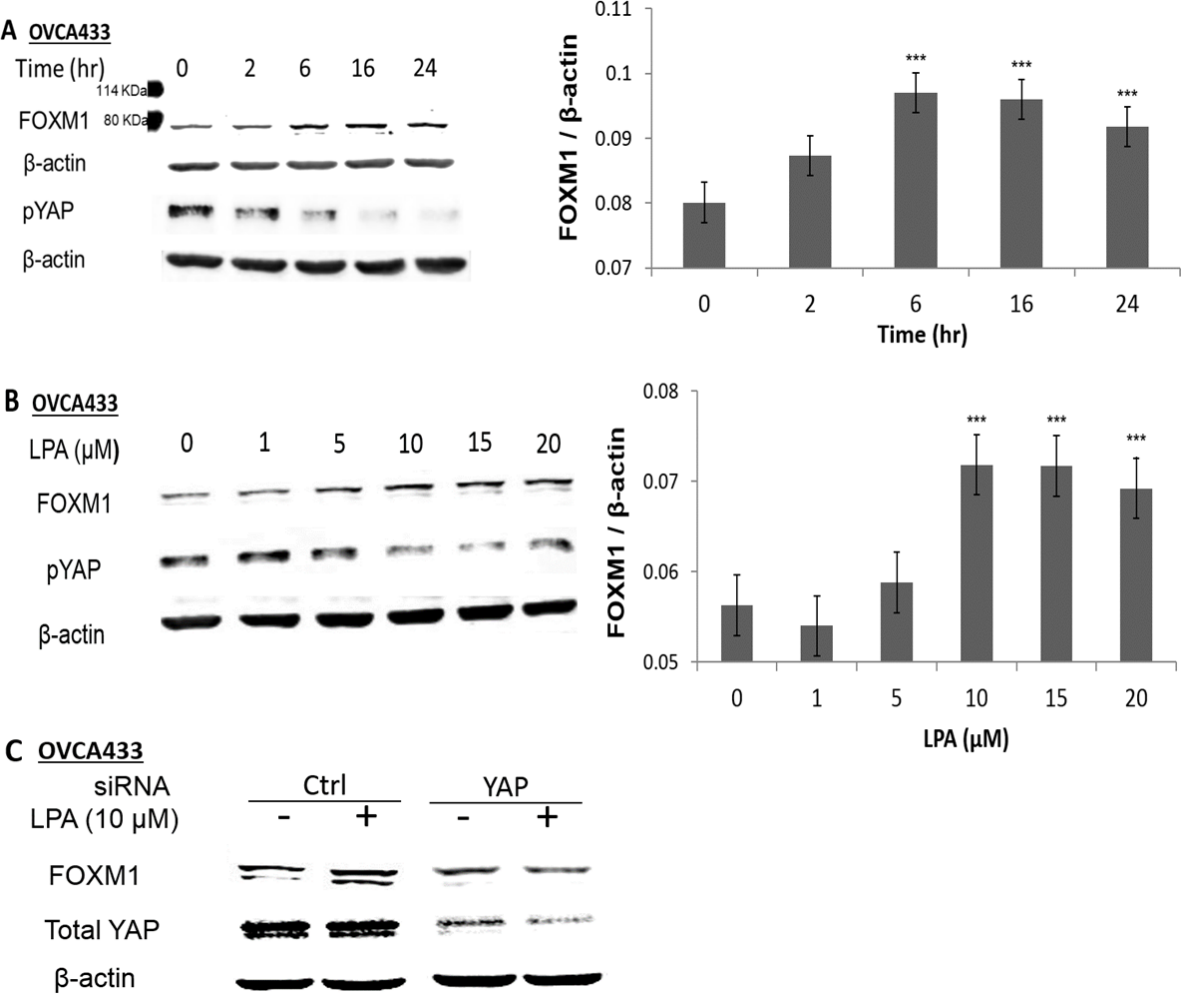
LPA induced FOXM1 up-regulation in EOC cells **A.** OVCA433 cells were starved for 16 hr prior to LPA (10 μM) treatment for different times as indicated. **B.** OVCA433 cells were starved for 16 hr prior to LPA treatment (6 hr) with different concentrations as indicated. The bar figures on the right panels are the summary of three independent experiments. **C.** OVCA433 cells were transfected with siRNA against YAP. The down-regulation of YAP and the effect on LPA-induced FOXM1 are shown. The antibody used to detect FOXM1 was from Sigma (Cat. Log # AV39518). *P < 0.05; **P < 0.01 and ***P < 0.001.

The potential regulation of FOXM1 by GPCRs and their ligands were tested using LPA activation, since it is a potent inducer of GPCR signaling pathways and a well-known oncolipid of EOC. LPA dose- and time-dependently up-regulated FOXM1 in OVCA433 cells, with 10 μM of LPA and 6 hr incubation as the optimal conditions for the induction (Figures 1A and 1B). This effect inversely correlated to LPA’s effect on pYAP. LPA-induced de-phosphorylation of YAP (dpYAP and activation of YAP) occurred prior to FOXM1 up-regulation (starting at 2 hr, Figure 1A) [18]. The quantitative assay results of three repeated experiments are shown in the bar format in Figures 1A and 1B. YAP was successfully down-regulated by specific siRNA (Figure 1C), which blocked LPA-induced FOXM1 up-regulation, indicating that YAP was functionally involved in LPA-induced FOXM1 up-regulation.

### LPA_1–3_ receptors and both of the PI3K and Rho pathways were involved in LPA-induced FOXM1 up-regulation

**Figure 2:**
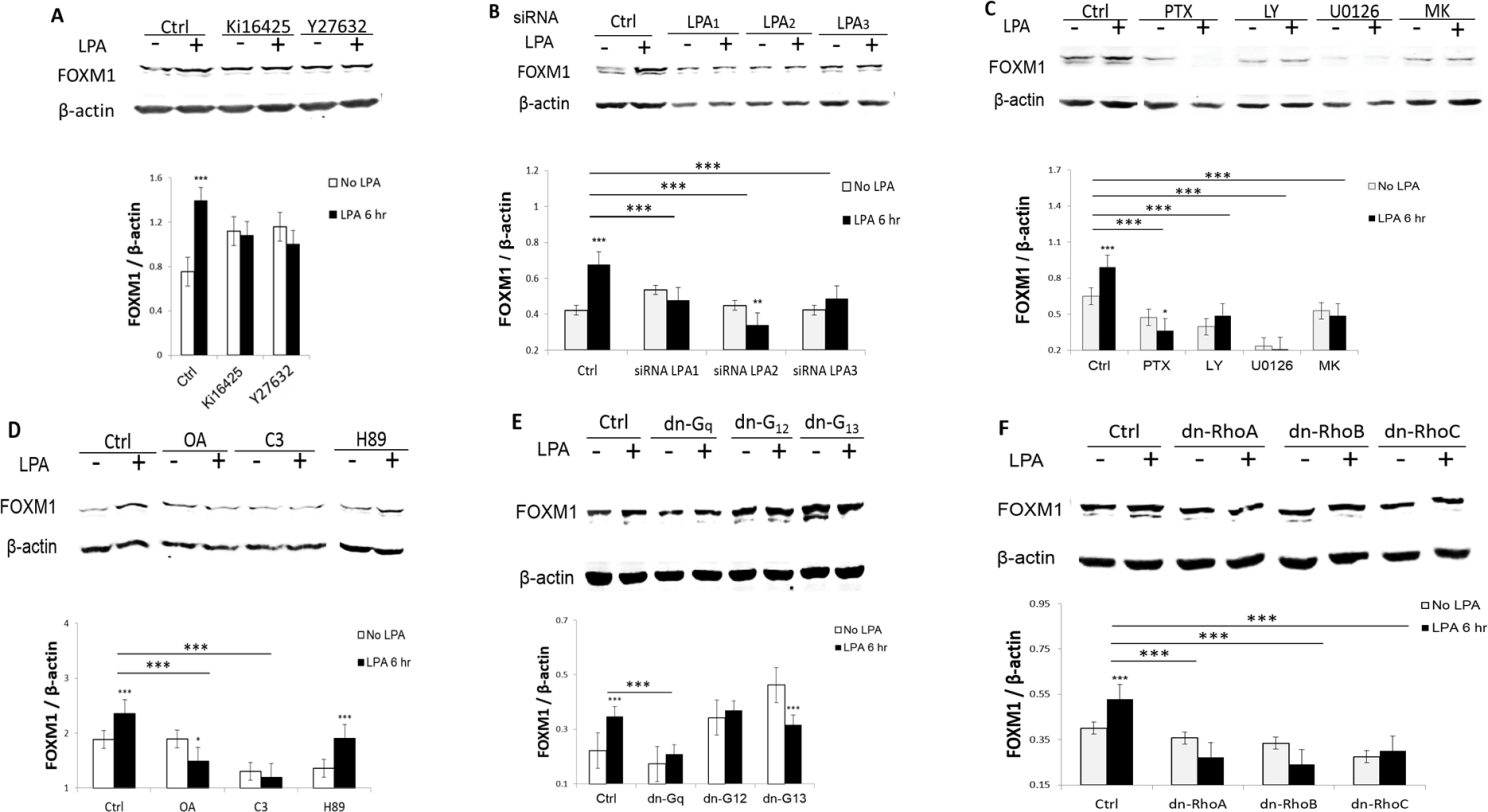
LPA_1–3_ and both of the PI3K and Rho pathways were involved in LPA-induced FOXM1 **A.** Pre-starved OVCA433 cells were treated with Ki16425 (10 μM) or Y27632 (10 μM) for 1 hr prior to LPA treatment (10 μM, 6 hr). FOXM1 was analyzed by Western blot. **B.** LPA (10 μM, 6 hr)-induced FOXM1 up-regulation were determined in LPA receptor specific siRNA-treated cells (48 hr post-transfection). **C.** Pre-starved OVCA433 cells were pretreated with PTX (100 ng/mL) for 16 hr; LY294002 (10 μM), U0126 (10 μM), or MK2203 (1 μM) for 1 hr, before LPA treatment for (10 μM, 6 hr). **D.** OVCA433 cells were starved overnight (16 hr), pretreated by OA(100 nM), C3(1 μg/mL), H89 (10 μM) for 1 hr, before LPA treatment for (10 μM, 6 hr). FOXM1 was detected using the Sigma antibody (Cat. Log # AV39518). The experiments were repeated at least three times. **E.** and **F.** OVCA433 cells were transfected with dominant negative plasmids for 48 hr, starved and then treated with LPA (10 μM for 6 hr). Cell lysates were analyzed by Western blot. Representative results are shown. *P < 0.05; **P < 0.01; and ***P < 0.001.

To identify the signaling pathways involved in LPA-induced FOXM1 expression, we first tested the effect of Ki16425, a selective inhibitor for LPA receptors LPA_1_/LPA_3_ and Y27632, a selective inhibitor of the Rho-kinase (ROCK). As shown in Figures 2A and 2B, although these two inhibitors increased the basal levels of FOXM1 (possibly due to an unknown non-specific effect), they blocked the LPA-induced up-regulation of FOXM1. To test the potential involvement of LPA receptors more specifically, we used siRNAs to block LPA_1_, LPA_2_, and LPA_3_, respectively. The efficacy and specificity of these siRNAs have been shown in our recent publication [18]. The results shown in Figure 2B suggest that all three LPA receptors are involved in LPA’s action on FOXM1.

LPA receptors activate G_i_, G_q_, and G_12/13_ [26]. We found that pertussis toxin (PTX, 100 ng/mL), a selective inhibitor of G_i_ protein; LY294002, a selective inhibitor of PI3K (LY, 10 μM); U0126 (10 μM), a selective inhibitor of the kinase of ERK1/2; and MK2203 (MK, 1 μM), a selective inhibitor of AKT, significantly inhibited both the basal and the LPA-induced FOXM1 expression (Figure 2C), suggesting the G_i_-PI3K-ERK-AKT pathway is involved in LPA-induced FOXM1 up-regulation.

LPA is also a potent inducer of activation of G_12/13_ and we have shown that the G_12/13_-Rho-ROCK-protein phosphatase 1A (PP1A) pathway is involved in LPA-induced YAP activation in OVC433 cells [26]. Hence, we tested a few blockers in this pathway. As expected, both okadaic acid (OA; 100 nM), an inhibitor of PP1A, and the Rho inhibitor C3 transferase (C3, 1 μg/mL) blocked LPA-induced FOXM1 expression (Figure 2D). In contrast, H89 (10 μM), an inhibitor of protein kinase A (PKA), a downstream effector of G_s_, did not block the LPA’s inducible effect (Figure 2D).

The effects of dominant forms of G_q_, G_12_, and G_13_ were tested. G_13_ was involved in LPA’s induction of FOXM1. On the other hand, G_12_ and G_q_ were much less or not involved (Figure 2E). Among small G proteins, RhoA and RhoB were likely to be involved (Figure 2F). These experiments were repeated at least three times and similar results were obtained.

### LPA regulated FOXM1 at the transcriptional level

**Figure 3:**
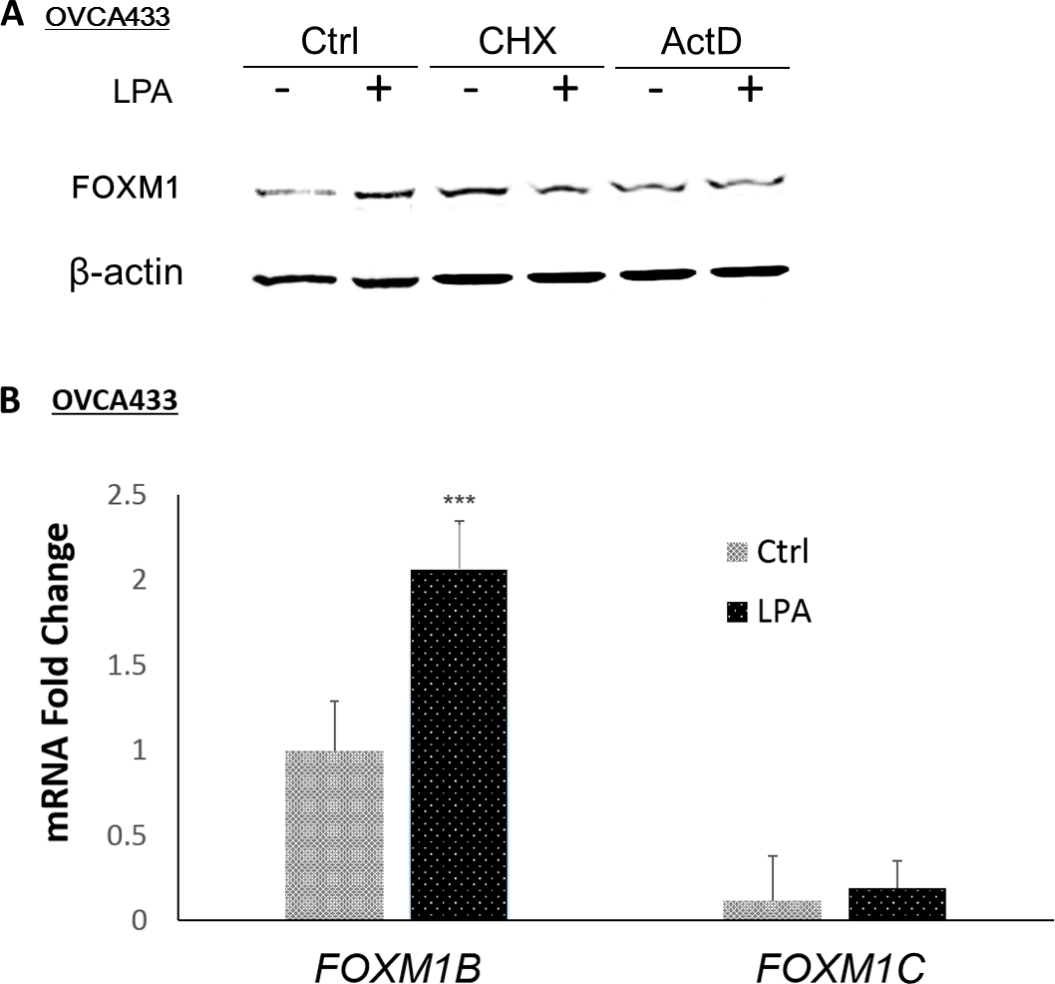
LPA-transcriptionally regulated FOXM1B in OVCA433 cells **A.** The transcriptional inhibitor ActD (1 μg/mL, 1 hr pre-treatment) and the translational inhibitor CHX (20 μg/mL, 1 hr pre-treatment) inhibited LPA-induced FOXM1 up-regulation by LPA (10 μM, 6 hr). FOXM1 was detected using the Sigma antibody (Cat. Log # AV39518). **B.** The mRNA levels of FOXM1 isoforms in OVCA433 cells with or without LPA (10 μM, 6 hr) treatment. FOXM1A was not detectible after 45 cycles of PCR amplification. Real-time RT-PCR conditions were described in Methods. *P < 0.05; **P < 0.01; and ***P < 0.001.

Human *FOXM1* is expressed in three distinct splice variants *FOXM1A, FOXM1B*, and *FOXM1C* [3]. These FOXM1 isoforms display the same DNA-binding specificity and bind to DNA-binding sites with the consensus sequence 5′-A-C/T-AAA-C/T-AA-3′. While FOXM1A is a transcriptional suppressor, FOXM1B and FOXM1C are functionally similar and both are transcriptional activators [12]. LPA-induced FOXM1 was blocked by both actinomycin D (ActD, 1 μg/mL) and cyclohexamine (CHX, 20 μg/mL) in OVCA433 cells (Figure 3A), suggesting that the regulation is at the transcriptional and translational levels. Quantitative RT-PCR (Q-PCR) analyses using three pairs of PCR primers specific for *FOXM1A, FOXM1B*, and *FOXM1C* were conducted. In OVCA433 cells, *FOXM1A* was not detectable (after 45 cycles’ amplification). *FOXM1C* was expressed at low levels, which were not significantly up-regulated by LPA (*P* > 0.05). In contrast, *FOXM1B* was the major isoform that was expressed in OVCA433 cells and was up-regulated by LPA (Figure 3B).

### FOXM1 was functionally involved in cell proliferation, migration, and invasion in EOC cells

**Figure 4:**
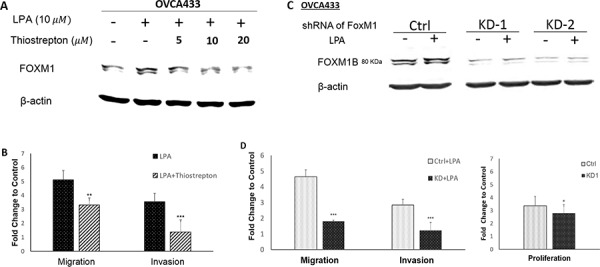
FOXM1 was functionally involved in cell proliferation, migration, and invasion in EOC cells OVCA433 cells were starved and pretreated with different doses of thiostrepton (a selective inhibitor of FOXM1) for 1 hr prior to LPA treatment (10 μM, 6 hr). Thiostrepton dose-dependently reduced LPA-induced FOXM1 expression **A.** and inhibited LPA-induced cell migration and invasion in OVCA433 cells **B.** FOXM1 down-regulation by shRNA **C.** reduced cell migration and invasion induced by LPA and cell proliferation induced by 2% FBS **D.** The results are from three independent experiments. FOXM1 was detected using the Sigma antibody (Cat. Log# AV39518). *P < 0.05; **P < 0.01; and ***P < 0.001.

The potential functional involvement of FOXM1 in proliferation, migration, and invasion was first tested using thiostrepton, a FOXM1 inhibitor. Thiostrepton dose-dependently reduced LPA-induced FOXM1 expression in OVCA433 cells (Figure 4A), which was accompanied by reduced cell migration and invasion in these cells (Figure 4B). To confirm these effects were indeed FOXM1 mediated, we established shRNA-FOXM1 knock-down (KD) stable OVCA433 cell lines (KD-1 and KD-2; Figure 4C). Cell migration and invasion induced by LPA were inhibited in FOXM1 KD cell lines. In addition, FBS-induced cell proliferation was reduced in FOXM1-KD1 cell line (Figure 4D).

### LPA-induced FOXM1 in additional EOC cell lines and FOXM1C was the dominant form in CAOV3 cells

**Figure 5:**
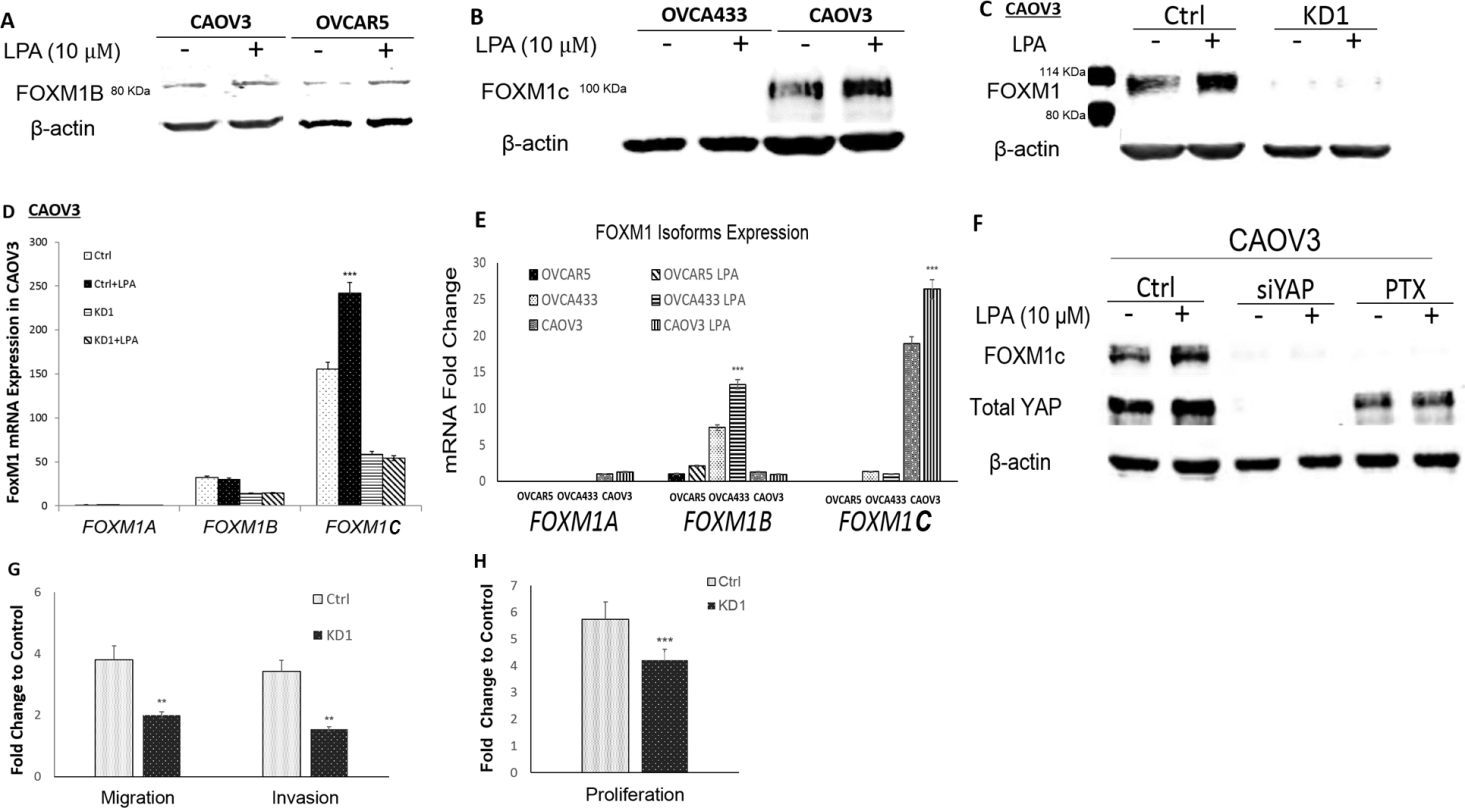
LPA-induced FOXM1 in other EOC cell lines and FOXM1C was the dominant form in CAOV3 cells **A.** LPA (10 μM and 6 hr) induced FOXM1 up-regulation in CAOV3 and OVCAR5 cells. CAOV3 and OVCAR5 cells expressed the FOXM1B with apparent MW of 80 KDa (Sigma, Cat. Log # AV39518). **B.** OVCA433 cells did not express FOXM1C, while CAOV3 cells expressed the FOXM1C with apparent MW of 100 KDa (Cell Signaling, Cat. Log # D12D5). **C.** CAOV3-FOXM1-KD cell line was established using shRNA against FOXM1 (detailed in Methods). The parental or control-shRNA transfected CAOV3 cells responded to LPA (10 μM, 6 hr) for FOXM1 up-regulation, which was blocked by KD-FOXM1. **D.** The dominant splicing form of FOXM1 in CAOV3 was FOXM1C. **E.** Comparison of the relative mRNA expression levels FOXM1 isoform in three EOC cell lines. **F.** KD-YAP and PTX inhibited LPA-induced FOXM1 up-regulation in CAOV3 cells. **G.** KD-FOXM1 inhibited LPA-induced cell migration and invasion. **H.** KD-FOXM1 inhibited FBS (2%)-induced cell proliferation. *P < 0.05; **P < 0.01; and ***P < 0.001.

LPA’s effect in FOXM1 was not limited to OVCA433 cells. As shown in Figure 5A, LPA (10 μM and 6 hr) treatment also induced FOXM1 up-regulation in CAOV3 and OVCAR5 cells. We used an anti-FOXM1 antibody from Sigma (Cat. Log # AV39518) to detect FOXM1 (∼80 KDa) in OVCA433 cells as shown in Figures 1–4 and Figure 5A. In the course of the study, we also used the anti-FOXM1 (D12D5, Cat. Log# 5436S) from Cell Signaling (Danvers, MA). Surprisingly, this antibody did not detect FOXM1 in OVCA433 cells, but detected a strong band in CAOV3 cells with an apparent molecular weight of ∼100 KDa (Figure 5B).

FOXM1B and FOXM1C are 748 and 763 amino acid proteins, respectively. Our results showed that Sigma antibody only detected FOXM1B with an apparent MW ∼80 KDa and the Cell Signaling antibody detected FOXM1C with an apparent MW ∼100 KDa (the increased MW may be contributed by both the increased amino acids and post-translational modifications) (Figure 5C). shRNA-mediated knock-down of FOXM1 in OVCA433 (Figure 4C) and CAOV3 (Figure 5C) support that both of these bands are isoforms of FOXM1. To test this notion further, we conducted RT-Q-PCR analyses in these cells. As shown in Figure 5D, in contrast to OVCA433 cells, FOXM1C was the dominant isoform in CAOV3. In OVCAR5 cells, both *FOXM1A* and *1C* forms were undetectable (after 45 cycles of PCR amplification) and *FOXM1B* was the major form, which was expressed in a relatively lower level than that in OVCA433 cells. A summary of the relative expression levels of the three *FOXM1* isoforms in the three EOC cell lines is shown in Figure 5E.

Treatment with siRNA against YAP or PTX completely blocked the basal and LPA-induced FOXM1 expression in CAOV3 cells, suggesting that the G_i_ and YAP signaling pathways are both involved, similar to that in OVCA433 cells (Figure 5F). In addition, FOXM1 KD in CAOV3 also reduced LPA-induced cell migration and invasion, and FBS-induced cell proliferation in these cells (Figures 5G and 5H).

### FOXM1 was functionally involved in tumorigenesis *in vivo*

**Figure 6:**
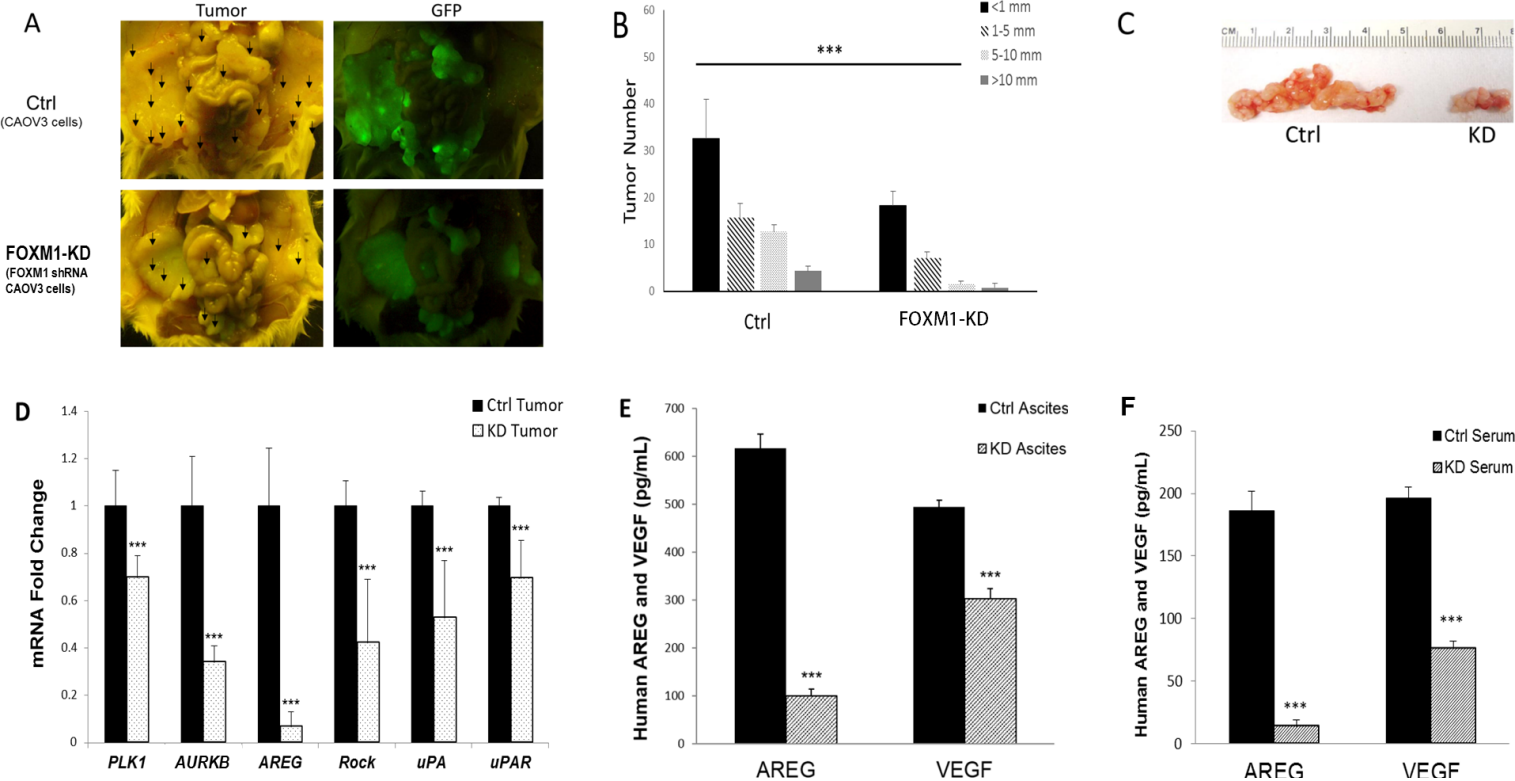
FOXM1 was functionally involved in tumorigenesis *in vivo* **A.** Representative pictures of the tumors formed in Ctrl or FOXM1-KD cells injected mice. 7–10 weeks old female NOD/SCID mice were i.p. injected with 5 × 10^6^ CAOV3 cells. Mice were euthanized 30 days after tumor cell injections and tumor development was analyzed. **B.** Tumor numbers and sizes were measured and compared. **C.** Effect of FOXM1-KD on CAOV3 generated ovarian tumor in NOD/SCID mice. **D.** FOXM1 related target genes were down-regulated at the mRNA level in tumors from CAOV3-KD injected mice, when compared to those in tumors from control cells. **E.** Human AREG and VEGF were reduced in ascites, measured by ELISA **F.** Human AREG and VEGF were reduced in serum, measured by ELISA *P < 0.05, **P < 0.01, ***P < 0.001.

We tested the roles of FOXM1 *in vivo* by i.p. injection of control-shRNA and FOXM1-shRNA transfected CAOV3 cells into NOD-SCID mice. The results are summarized in Figure 6. While all mice formed tumors, FOXM1 KD significantly reduced the tumor and ascites load in the mice. Figure 6A shows the representative pictures of the tumors formed in the control- and FOXM1-KD cell-injected mice. The tumor nodule numbers and sizes, and the tumors grew on the ovaries in particular, as well as ascites volumes were significantly reduced in FOXM1-KD cell injected mice, compared to control mice (Figures 6B and 6C). In addition, more organ sites were affected in control mice (Table 1, *n* = 5 mice in each group), suggesting that the reduced cellular activities (proliferation, migration, and invasion) in FOXM1-KD cells detected *in vitro* are likely contributing to the reduced tumoirgenesis *in vivo*.

**Table 1:** Summary of ascites volume and metastasis invaded organs

Injected cells	No. of Mice	Ascites Incidence	Ascites Volume (mean)	Tumor Incidence	Invaded organs sites
CAOV3	5	3/5	0.2–0.4 ml	5/5	D.L.M.O.SI.W (all 5/5)
FoxM1-KD CAOV3	5	1/5	0.1 ml	5/5	D (4/5) W (4/5) M (2/5)

D, diaphragm; L, liver; M, mesentery; O, omentum; SI, small intestine; W, peritoneal wall.

We also determined and compared the levels of several FOXM1 targets in the blood, ascites, and/or tumors of the control- and FOXM1-KD cell-injected mice. FOXM1 regulates the expression of over 220 genes [31]. We analyzed mRNA expression or protein secretion levels of several representative genes involved in proliferation, cell cycling, migration, or invasion, including: amphiregulin (ARGE), polo-like kinase 1 (PLK1), aurora kinase B (AURKB), Rho-associated coiled-coil containing protein kinase 1 (ROCK1), uPA (plasminogen activator, urokinase; gene name PLAU), uPAR (plasminogen activator, urokinase receptor; gene name PLAUR), and vascular endothelial growth factor A (VEGFA) in paired tumors from the control shRNA-transfected and FOXM1-KD cells derived tumors. As shown in Figure 6D, PLK1, AURKB, AREG, ROCK1, uPA and uPAR were significantly down regulated in FOXM1-KD derived tumors. In addition, AREG and VEGF secreted in ascites and serum were significantly down regulated in KD cell injected mice (Figures 6E and 6F). Since human primers and ELASA kits were used, these changes in gene or protein levels are human tumor cell derived changes.

## DISCUSSION

We show here, for the first time, that LPA is capable of regulating FOXM1 expression in EOC cells, which has not been shown in any other cell types. Interestingly, this regulation is mediated by both the G_i_-PI3K and G_12/13_-Rho-YAP pathways. In one of our previous publications, we used pharmacological inhibitors and genetic approaches to conduct detailed mechanistic studies of LPA and S1P-induced Akt activation in ovarian cancer cells. We have shown that the kinase activity and S473 phosphorylation of Akt induced by LPA and S1P requires both mitogen-activated protein (MAP) kinase (MEK) and p38 MAP kinase. MEK is likely to be upstream of p38 in the 9 cell lines examined [32]. Other than one report in malignant mesothelioma (MM) cells [16], this is the first evidence that YAP is an important up-stream regulator of FOXM1 in epithelial cancer cells. The Hippo-YAP pathway has emerged as a critical oncogenic pathway in recent years [17–19]. Our work has linked the oncolipid LPA, the oncogenic YAP pathway, and the FOXM1 network (which has been shown to be altered in 84% cases of HGSC) in EOC cells, providing a base for developing novel therapeutics for EOC.

Our results suggest that each of the three LPA receptors, LPA_1–3_, is essential for the action of LPA. Ki16425, a selective antagonist of LPA_1_/LPA_3_ also completely blocked the effect of LPA. In addition LPA_2_ was also involved. We have previously shown that LPA receptors form homo- and hetero-dimers within the LPA receptor subgroup [33]. The results in this work suggest that these dimers may be functionally involved in LPA’s actions. Hence, each of them is essential for LPA’s action.

FOXM1 is a key regulator of over 200 genes involved in cancers [31]. We analyzed mRNA expression or protein secretion levels of several representative genes involved in proliferation and cell cycling, including ARGE, PLK1, AURKB, as well as several others likely to be involved in cell migration and/or invasion, such as ROCK1, uPA, uPAR, and vascular endothelial growth factor A (VEGFA), in paired tumors from the control shRNA-transfected and FOXM1-KD cell derived tumors. All of these genes/proteins have already been linked to tumor development of EOC. Other than the well-known functions of AREG, uPA, uPAR, and VEGF, aurora kinases B was not detected in non-neoplastic ovaries (*n* = 18), but almost all (79/80; 99%) ovarian carcinomas exhibited aurora-B positive tumor cells [34]. PLK1 and ROCK1 are also over-expressed in malignant ovarian tissues and/or functionally involved in EOC [35–37]. These results support not only the specific effects on FOXM1 in FOXM1-KD cells, but also on the molecular basis to target the FOXM1 network in EOC.

FOXM1 is a typical proliferation-associated transcription factor that is intimately involved in tumorigenesis. The important roles of FOXM1 in EOC have been implicated in previous studies [1, 2, 4–6]. Although the effect of a FOXM1 selective inhibitor, thiostrepton, has been tested in *in vivo* [5], the concept of targeting FOXM1 in EOC needs to be tested further since the mouse model and reagents used may improperly address the targeting issues related to FOXM1, due to the potential off-target effect of the inhibitor, the non HGSC nature of the cell line used (A2780 or SKOV3); and the route of the injection (s.c.) [5, 14]. In addition, specific tumor cell targeting may be necessary.

Our studies using shRNA-mediated KD of FOXM1 in EOC cells have more specifically demonstrated the role of FOXM1 in cell proliferation, migration, and invasion *in vitro* and tumorigenesis *in vivo*. While pharmacological inhibitors are most commonly used for cancer treatment, all inhibitors have off targets. Our *in vitro* signaling results suggest that although several inhibitors used blocked the LPA-induction effects, they may increase the basal levels of FOXM1, which is an unwanted effect. Moreover, inhibitors also target host cells, which may result in toxicity. In recent years, other forms of therapeutics have been moving into clinical practice, including neutralizing antibodies and shRNA-mediated specific gene down regulation.

The three EOC cells lines used in this work may represent HGSC better than other EOC cell lines used in previous FOXM1 work. Recently, the most commonly used EOC cell lines, including A2780 and SKOV3, have been considered as non-HGSC cell lines [14, 38] based on genetic and functional characterizations. CAOV3 cells are considered “likely to be HGSC” [9]. OVCA433 and OVCAR5 cells are not included in the two sets analyses, but these cells have been shown to be serous cancer cell lines with a dysfunctional p53 pathway [39, 40], one of the most commonly shared features of HGSC cells. In addition, we used the i.p. injection (vs. the s.c. model in the previous studies using A2780 cells) [5] model in the present work to better recapitulate EOC metastatic disease.

Human FOXM1 is expressed in three distinct splice variants, which arise from the same gene through differential splicing of the two facultative exons A1 and A2 [31]. The resulting proteins are 801, 748, and 763 for FOXM1A, 1B and 1C, respectively. While FOXM1A is transcriptionally inactive in most cells, both FOXM1B and FOXM1C are biologically active, and they play similar biological roles. However, their expression patterns are cell type-dependent and their regulations and biding partners may differ [31]. FOXM1C has been found to be the predominant form expressed in pancreatic tumors and cancer cell lines [41]. Interestingly, we have found that FOXM1 isoform expression pattern is cell line-dependent. Both the FOXM1B and FOXM1C forms were functionally involved in cell proliferation, migration, and invasion. In addition, LPA was able to up-regulate both forms in a cell line-dependent manner. Unexpected, the Sigma and Cell Signaling FOXM1 antibodies appear to only recognize the 1B or the 1C form, respectively (Figure 5B), even though both antibodies should recognize both forms theoretically. The immunogen peptide (KTSPRRPLILKRRRLPLPVQNAPSETSEEEPKRSPA QQESNQAEASKEVA) used for generating the Sigma antibody is present in both 1B and 1C forms (the first 51 AA in both forms). The Cell Signaling FOXM1 antibody is a monoclonal antibody against human recombinant FOXM1 protein, which typically detects FOXM1 as a 100 kDa protein. Although this antibody is predicted to recognize FOXM1B (Cell Signaling Data sheet), we did not detect any bands between 50–110 KDa in OVCA433 cells (Figure 5B), suggesting that even if it might be recognizing FOXM1B, its sensitivity is low. While the reasons why these antibodies showed 1B and 1C specificity are unclear, they are useful in isoform specificity studies.

Taken together, our data support the development of new EOC therapeutics centering at the FOXM1 network, including identified up-stream regulators and down-stream targets. Tumor cell specific targeting may be necessary and tumor and or tumor-host interaction generated secreted factors, such as AREG and/or VEGF may be used as follow-up factors to monitor treatment and diseases progression.

## MATERIALS AND METHODS

### Reagents

Oleoyl-LPA was purchased from Avanti Polar Lipids (Birmingham, AL). The following inhibitors or reagents were used in this study: LY294002 (Enzo Life Sciences, Farmingdale, NY); MK2206 and Y27632 (Biovision, Milpitas, CA); C3 exoenzyme (Cytoskeleton, Denver, CO); Ki16425 and okadaic acid (OA; Calbiochem, San Diego, CA); pertussis toxin (PTX; Invitrogen, Grand Island, NY); and actinomycin D (ActD) and cyclohexamide (CHX; Sigma-Aldrich, St. Louis, MO). Alexa fluor-conjugated secondary antibodies were from Life Technologies, Grand Island, NY. The dominant negative (dn) and constitutive active (ca) forms of large and small G protein constructs were from UMR cDNA Resource Center (Rolla, MO). The antibody mainly against FOXM1b was purchased from Sigma (Cat. Log. # AV39518). The antibodies against phospho-YAP (Ser127) and FOXM1C (D12D5, Cat. Log# 5436S) were purchased from Cell Signaling (Danvers, MA). The antibody against Total-YAP was purchased from Santa Cruz Biotechnology (Santa Cruz, CA).

### Cell lines and culture

The OVCA433 cell line was a kind gift of Dr. R. Bast (M.D. Anderson); the CAOV3 and the OVCAR5 cells were obtained from ATCC (Manassas, VA). All cell lines were maintained in a humidified atmosphere at 37°C with 5% CO_2_. OVCA433 cells were cultured in RPMI 1640 with glutamine, 10% FBS (ATCC, Manassas, VA), and 100 μg/mL penicillin/streptomycin (P/S). CAOV3 were cultured in DMEM with glutamine, 10% FBS (ATCC, Manassas, VA), and 100 μg/mL P/S. OVCAR5 cells were cultured in DMEM with glutamine, 10% FBS (ATCC, Manassas, VA), 0.1 mM MEM Non-Essential Amino Acids (NEAA), 2 mM L-glutamine and 100 μg/mL P/S. For serum starvation, cells were incubated in the basal medium without FBS or antibiotics. LPA treatment was always in cells starved from serum for 16 hr.

### Western blot analysis

Western blot analyses were conducted using standard procedures and proteins were detected using primary antibodies and fluorescent secondary antibodies (IRDye 800CW-conjugated or IRDye 680-conjugated anti-species IgG, Li-Cor Biosciences, Lincoln, NE). The fluorescent signals were captured on an Odyssey Infrared Imaging System (Li-Cor Biosciences, Lincoln, NE) with both 700- and 800-nm channels. Boxes were manually placed around each band of interest, and the software returned near-infrared fluorescent values of raw intensity with background subtraction (Odyssey 3.0 analytical software, Li-Cor Biosciences, Lincoln, NE). The protein MW marker used was the Prestained SDS-PAGE Standards, broad range (BIO_RAD, Cat. Log # 161–0318).

### DNA and RNA transfection

Plates (6-well) were seeded with 5 × 10^4^ cell/well in 2 mL media 24 hr before transfection; cells were 80%–90% confluent and were transfected with siRNA (100 pmol/well) or plasmid DNA (4 μg/well) using Lipofectamine 2000 Reagent (Life Technologies, Grand Island, NY) according to manufacturer’s instruction. After 48 hr of transfection, cells were starved for migration and invasion assays. All siRNAs were purchased from Santa Cruz Biotechnology (Santa Cruz, CA).

### Establish stable clones and drug selection concentrations

*FOXM1* shRNA lentiVirus vectors with GFP (OriGene, Rockville, MD) were transfected to 293T cells for virus packaging. OVCA433 or CAOV3 cells were infected by virus 3 times and stable clones were selected by puromycin (concentration 0.5 μg/mL) for 10 days.

### Reverse-transcription quantitative real-time PCR

Cells were washed with cold PBS and collected in the Qiagen RLT lysis buffer (Qiagen, Valencia, CA). RNA was extracted with the RNeasy mini kit (Qiagen, Valencia, CA) and reverse transcribed by M-MLV reverse transcriptase. Quantitative real-time PCR was performed on a Light Cycler 480 (Roche, Indianapolis, IN) with a SYBR Green I Master Mix (Roche, Indianapolis, IN). mRNA abundance was normalized to GAPDH. Negative controls contained no reverse transcription or the reverse transcriptase. RNAs from triplicate cell pellets per condition were analyzed. Relative gene expression was calculated using the method given in Applied Biosystems User Bulletin No.2 (P/N 4303859B), with non-targeting siRNA-treated cells acting as the control in each data set. Primer pairs used in this study were: GAPDH: F, 5′-CACCATTGGCAATGAGCGGTTC-3′/R, 5′-AGGTCTTTGCGGATGTCCACGT-3′; FOXM1A: F, 5′-TGGGGAACAGGTGGTGTTTGG-3′/R, 5′-GCTAG CAGCACTGATAAACAAAG-3′; FOXM1B: F, 5′-CCAG GTGTTTAAGCAGCAGA-3′/R, 5′-TCCTCAGCTAGCA GCACCTTG-3′; FOXM1C: F, 5′-CAATTGCCCGAGCA CTTGGAATCA-3′/R, 5′-TCCTCAGCTAGCAGCACC TTG-3′. PLK1: F, 5′-CTCCTGGAGCTGCACAAGAG GAGGAA-3′/R, 5′-TCTGTCTGAAGCATCTTCTGGA TGAG-3′; AURKB: F, 5′-CTGCCATGGGAAGAAGG TGATTCA-3′/R, 5′-GATGCGGCGATAGGTCTCGTT G-3′; AREG: F, 5′-GTGGTGCTGTCGCTCTTGATA-3′/R, 5′-ACTCACAGGGGAAATCTCACT-3′; ROCK1: F, 5′-GACCTGTAACCCAAGGAGAT-3′/R, 5′-GGAAAG TGGTAGAGTGTAGG-3′. uPA: F, 5′-CACGCAAGGGG AGATGAA-3′/R, 5′-ACAGCATTTTGGTGGTGACTT-3′. uPAR: F, 5′-CAACGACACCTTCCACTTC-3′/R, 5′-GCA CAGCCTCTTACCATATAG-3′.

### Migration assays

To measure cell migration, the undersurfaces of Transwells (Costar, Corning, NY) were coated with collagen IV (10 μg/mL, Upstate Biotechnology, Lake Placid, NY) at 4°C overnight. Coated transwells were then placed into a 24-well plate containing 0.5 mL of serum-free medium. To determine the effect of FoxM1 in LPA induced migration, cells were starved and pretreated with thiostrepton (10 μM) prior to treatment with LPA (10 μM, 6 hr); detached cells (by PBS containing 10 mM EDTA) were washed several times with serum-free medium. Cells (10^6^ in 100 μL) in serum-free medium were added to the upper chamber in each transwell and allowed to migrate for 4 hr at 37°C. LPA (10 μM) were added to the lower chamber as the chemoattractant. Cotton swabs were used to remove cells in the upper surface of the transwells, and migratory cells attached on the undersurface were stained with crystal violet solution. Transwells were rinsed with water and air-dried. Crystal violet-stained attached cells were solubilized in 100 μL of 10% acetic acid and quantitated using a microplate reader at 600 nm.

### Invasion assays

Inner side of Transwells (8 μM pore size) were coated with Matrigel at a concentration of 1.1 μg/mL and placed in a modified Boyden chamber. Serum starved (24 hr) cells were treated w/o LPA (10 μM, 6 hr), then trypsinized and 1.5 × 10^5^ cells/well were added to the top chamber. LPA (10 μM) served as a chemoattractant and was added to the lower chambers. Cells were allowed to invade through the Matrigel barrier for 24 hr. After incubation, filters were fixed and stained in Diff-Quick staining solutions. Non-invading cells were removed using a cotton swab while invading cells on the underside of the filter were enumerated using an inverted microscope. All experiments were performed in triplicate and repeated at least three times.

### Proliferation assay

Cells were seeded in 96-well plates (2000 cells/well), cultured for 48 hr, and then starved for 24 hr by replacing the media with serum-free media (RPMI 1640 for OVCA433 cells and DMEM for CAOV3 cells), followed by addition of 2% FBS, in the presence or absence of thiostrepton (10 μM). The cells were further cultured for 48 hr. Cell proliferation was evaluated by MTT hydrolysis using Cell Counting Kit-8 (Dojindo Molecular Technologies, Rockville, MA).

### ELASA assays

Ascites and serum were collected from NOD/SCID mice (either CAOV3 control or FOXM1-KD CAOV3 cells injected) and stored at −80°C until ELISA assays were conducted. ELISA assays were performed using ELASA kits from the Human ELISA Development Systems (R&D Systems. Minneapolis, MN) in triplicate wells according to the manufacturer’s instructions. The optical density at 450 nm was measured on an automated plate reader (PerkinElmer, Santa Clara, CA). Experiments were repeated three times.

### Xenograft mouse model

Female NOD/SCID mice were obtained from the In vivo Therapeutics Core, Indiana University School of Medicine (Indianapolis, IN) at 7 to 10 weeks of age. CAOV3 cells (both FOXM1-KD and control cells, 5 × 10^6^ in 500 μL of PBS) were i.p. injected into mice. Tumors were monitored in living mice every day. Mice were euthanized 30 days after tumor cell injections, and tumor development was analyzed. Tumors were counted at each metastatic location, and tumor diameters were measured. Ascites were collected from NOD/SCID mice and, after centrifugation, floating living tumor cells (GFP-expressing cells) were counted. Animal protocols were approved by the Indiana University School of Medicine Animal Care and Use Committee.

### Statistical analyses

The Student’s *t*-test was utilized to assess the statistical significance of the difference between two treatments. The asterisk rating system as well as quoting the *P* value in this study was *P < 0.05; **P < 0.01; and ***P < 0.001. A *P* value of less than 0.05 was considered significant.

## References

[1] Cancer Genome Atlas Research N (2011). Integrated genomic analyses of ovarian carcinoma. Nature.

[2] Nikitin AF, Afotey AO, Nikitin AY Role of the stem cell niche in the pathogenesis of epithelial ovarian cancers. Molecular & Cellular Oncology.

[3] Wierstra I (2013). FOXM1 (Forkhead box M1) in tumorigenesis: overexpression in human cancer, implication in tumorigenesis, oncogenic functions, tumor-suppressive properties, and target of anticancer therapy. Advances in cancer research.

[4] Elgaaen BV, Olstad OK, Sandvik L, Odegaard E, Sauer T, Staff AC, Gautvik KM (2012). ZNF385B and VEGFA are strongly differentially expressed in serous ovarian carcinomas and correlate with survival. PloS one.

[5] Chan DW, Hui WW, Cai PC, Liu MX, Yung MM, Mak CS, Leung TH, Chan KK, Ngan HY (2012). Targeting GRB/ERK/FOXM1 signaling pathway impairs aggressiveness of ovarian cancer cells. PloS one.

[6] Zhao F, Siu MK, Jiang L, Tam KF, Ngan HY, Le XF, Wong OG, Wong ES, Gomes AR, Bella L, Khongkow P, Lam EW, Cheung AN (2014). Overexpression of Forkhead Box Protein M1 (FOXM1) in Ovarian Cancer Correlates with Poor Patient Survival and Contributes to Paclitaxel Resistance. PloS one.

[7] Bhat UG, Halasi M, Gartel AL (2009). FoxM1 is a general target for proteasome inhibitors. PloS one.

[8] Zhang L, Ging NC, Komoda T, Hanada T, Suzuki T, Watanabe K (2005). Antibiotic susceptibility of mammalian mitochondrial translation. FEBS letters.

[9] Kwok JM, Myatt SS, Marson CM, Coombes RC, Constantinidou D, Lam EW (2008). Thiostrepton selectively targets breast cancer cells through inhibition of forkhead box M1 expression. Molecular cancer therapeutics.

[10] Halasi M, Gartel AL (2009). A novel mode of FoxM1 regulation: positive auto-regulatory loop. Cell Cycle.

[11] Hegde NS, Sanders DA, Rodriguez R, Balasubramanian S (2011). The transcription factor FOXM1 is a cellular target of the natural product thiostrepton. Nature chemistry.

[12] Lok GT, Chan DW, Liu VW, Hui WW, Leung TH, Yao KM, Ngan HY (2011). Aberrant activation of ERK/FOXM1 signaling cascade triggers the cell migration/invasion in ovarian cancer cells. PloS one.

[13] Wen N, Wang Y, Wen L, Zhao SH, Ai ZH, Wang Y, Wu B, Lu HX, Yang H, Liu WC, Li Y (2014). Overexpression of FOXM1 predicts poor prognosis and promotes cancer cell proliferation, migration and invasion in epithelial ovarian cancer. J Transl Med.

[14] Domcke S, Sinha R, Levine DA, Sander C, Schultz N (2013). Evaluating cell lines as tumour models by comparison of genomic profiles. Nat Commun.

[15] Balli D, Zhang Y, Snyder J, Kalinichenko VV, Kalin TV (2011). Endothelial cell-specific deletion of transcription factor FoxM1 increases urethane-induced lung carcinogenesis. Cancer Res.

[16] Mizuno T, Murakami H, Fujii M, Ishiguro F, Tanaka I, Kondo Y, Akatsuka S, Toyokuni S, Yokoi K, Osada H, Sekido Y (2012). YAP induces malignant mesothelioma cell proliferation by upregulating transcription of cell cycle-promoting genes. Oncogene.

[17] Zhao B, Lei QY, Guan KL (2008). The Hippo-YAP pathway: new connections between regulation of organ size and cancer. Curr Opin Cell Biol.

[18] Cai H, Xu Y (2013). The role of LPA and YAP signaling in long-term migration of human ovarian cancer cells. Cell Commun Signal.

[19] Ma Y, Yang Y, Wang F, Wei Q, Qin H (2014). Hippo-YAP signaling pathway: A new paradigm for cancer therapy. Int J Cancer.

[20] Xu Y, Shen Z, Wiper DW, Wu M, Morton RE, Elson P, Kennedy AW, Belinson J, Markman M, Casey G (1998). Lysophosphatidic acid as a potential biomarker for ovarian and other gynecologic cancers. JAMA.

[21] Xu Y, Casey G, Mills GB (1995). Effect of lysophospholipids on signaling in the human Jurkat T cell line. J Cell Physiol.

[22] Kim KS, Sengupta S, Berk M, Kwak YG, Escobar PF, Belinson J, Mok SC, Xu Y (2006). Hypoxia enhances lysophosphatidic acid responsiveness in ovarian cancer cells and lysophosphatidic acid induces ovarian tumor metastasis *in vivo*. Cancer Res.

[23] Mills GB, Moolenaar WH (2003). The emerging role of lysophosphatidic acid in cancer. Nat Rev Cancer.

[24] Fang X, Schummer M, Mao M, Yu S, Tabassam FH, Swaby R, Hasegawa Y, Tanyi JL, LaPushin R, Eder A, Jaffe R, Erickson J, Mills GB (2002). Lysophosphatidic acid is a bioactive mediator in ovarian cancer. Biochimica et biophysica acta.

[25] Tanyi J, Rigo J (2009). Lysophosphatidic acid as a potential target for treatment and molecular diagnosis of epithelial ovarian cancers. Orv Hetil.

[26] Yung YC, Stoddard NC, Chun J (2014). LPA receptor signaling: pharmacology, physiology, and pathophysiology. J Lipid Res.

[27] Sengupta S, Xiao YJ, Xu Y (2003). A novel laminin-induced LPA autocrine loop in the migration of ovarian cancer cells. FASEB J.

[28] Ren J, Xiao YJ, Singh LS, Zhao X, Zhao Z, Feng L, Rose TM, Prestwich GD, Xu Y (2006). Lysophosphatidic acid is constitutively produced by human peritoneal mesothelial cells and enhances adhesion, migration, and invasion of ovarian cancer cells. Cancer Res.

[29] Sengupta S, Kim KS, Berk MP, Oates R, Escobar P, Belinson J, Li W, Lindner DJ, Williams B, Xu Y (2007). Lysophosphatidic acid downregulates tissue inhibitor of metalloproteinases, which are negatively involved in lysophosphatidic acid-induced cell invasion. Oncogene.

[30] Li H, Wang D, Zhang H, Kirmani K, Zhao Z, Steinmetz R, Xu Y (2009). Lysophosphatidic acid stimulates cell migration, invasion, and colony formation as well as tumorigenesis/metastasis of mouse ovarian cancer in immunocompetent mice. Molecular cancer therapeutics.

[31] Wierstra I (2013). The transcription factor FOXM1 (Forkhead box M1): proliferation-specific expression, transcription factor function, target genes, mouse models, and normal biological roles. Advances in cancer research.

[32] Baudhuin LM, Cristina KL, Lu J, Xu Y (2002). Akt activation induced by lysophosphatidic acid and sphingosine-1-phosphate requires both mitogen-activated protein kinase kinase and p38 mitogen-activated protein kinase and is cell-line specific. Molecular pharmacology.

[33] Zaslavsky A, Singh LS, Tan H, Ding H, Liang Z, Xu Y (2006). Homo- and hetero-dimerization of LPA/S1P receptors, OGR1 and GPR4. Biochimica et biophysica acta.

[34] Beussel S, Hasenburg A, Bogatyreva L, Hauschke D, Werner M, Lassmann S (2012). Aurora-B protein expression is linked to initial response to taxane-based first-line chemotherapy in stage III ovarian carcinoma. J Clin Pathol.

[35] Benoit DS, Henry SM, Shubin AD, Hoffman AS, Stayton PS (2010). pH-responsive polymeric sirna carriers sensitize multidrug resistant ovarian cancer cells to doxorubicin via knockdown of polo-like kinase 1. Mol Pharm.

[36] Weichert W, Denkert C, Schmidt M, Gekeler V, Wolf G, Kobel M, Dietel M, Hauptmann S (2004). Polo-like kinase isoform expression is a prognostic factor in ovarian carcinoma. Br J Cancer.

[37] Ohta T, Takahashi T, Shibuya T, Amita M, Henmi N, Takahashi K, Kurachi H (2012). Inhibition of the Rho/ROCK pathway enhances the efficacy of cisplatin through the blockage of hypoxia-inducible factor-1alpha in human ovarian cancer cells. Cancer Biol Ther.

[38] Beaufort CM, Helmijr JC, Piskorz AM, Hoogstraat M, Ruigrok-Ritstier K, Besselink N, Murtaza M, van IWF, Heine AA, Smid M, Koudijs MJ, Brenton JD, Berns EM, Helleman J (2014). Ovarian cancer cell line panel (OCCP): clinical importance of *in vitro* morphological subtypes. PloS one.

[39] Debernardis D, Sire EG, De Feudis P, Vikhanskaya F, Valenti M, Russo P, Parodi S, D’Incalci M, Broggini M (1997). p53 status does not affect sensitivity of human ovarian cancer cell lines to paclitaxel. Cancer Res.

[40] Creighton CJ, Fountain MD, Yu Z, Nagaraja AK, Zhu H, Khan M, Olokpa E, Zariff A, Gunaratne PH, Matzuk MM, Anderson ML (2010). Molecular profiling uncovers a p53-associated role for microRNA-31 in inhibiting the proliferation of serous ovarian carcinomas and other cancers. Cancer Res.

[41] Kong X, Li L, Li Z, Le X, Huang C, Jia Z, Cui J, Huang S, Wang L, Xie K (2013). Dysregulated expression of FOXM1 isoforms drives progression of pancreatic cancer. Cancer Res.

